# Development of a Wearable Cardiac Monitoring System for Behavioral Neurocardiac Training: A Usability Study

**DOI:** 10.2196/mhealth.5288

**Published:** 2016-04-22

**Authors:** Akib A Uddin, Plinio P Morita, Kevin Tallevi, Kevin Armour, John Li, Robert P Nolan, Joseph A Cafazzo

**Affiliations:** ^1^ Institute of Biomaterials and Biomedical Engineering Faculty of Medicine University of Toronto Toronto, ON Canada; ^2^ Centre for Global eHealth Innovation Techna Institute University Health Network Toronto, ON Canada; ^3^ Cardiac eHealth & Behavioural Cardiology Research Unit Toronto General Hospital University Health Network, University of Toronto Toronto, ON Canada; ^4^ Institute of Health Policy, Management and Evaluation Dalla Lana School of Public Health University of Toronto Toronto, ON Canada

**Keywords:** mobile health, smartphones, sensor devices and platforms, wireless technology, mobile applications, electrocardiography, biofeedback, psychology, blood pressure, stress, physiological, relaxation

## Abstract

**Background:**

Elevated blood pressure is one of the main risk factors for death globally. Behavioral neurocardiac training (BNT) is a complementary approach to blood pressure and stress management that is intended to exercise the autonomic reflexes, improve stress recovery, and lower blood pressure. BNT involves cognitive-behavioral therapy with a paced breathing technique and heart rate variability biofeedback. BNT is limited to in-clinic delivery and faces an accessibility barrier because of the need for clinical oversight and the use of complex monitoring tools.

**Objective:**

The objective of this project was to design, develop, and evaluate a wearable electrocardiographic (ECG) sensor system for the delivery of BNT in a home setting.

**Methods:**

The wearable sensor system, *Beat*, consists of an ECG sensor and a mobile app. It was developed iteratively using the principles of test-driven Agile development and user-centered design. A usability study was conducted at Toronto General Hospital to evaluate feasibility and user experience and identify areas of improvement.

**Results:**

The *Beat*sensor was designed as a modular patch to be worn on the user’s chest and uses standard ECG electrodes. It streams a single-lead ECG wirelessly to a mobile phone using Bluetooth Low Energy. The use of small, low-power electronics, a low device profile, and a tapered enclosure allowed for a device that can be unobtrusively worn under clothing. The sensor was designed to operate with a mobile app that guides users through the BNT exercises to train them to a slow-paced breathing technique for stress recovery. The BNT app uses the ECG captured by the sensor to provide heart rate variability biofeedback in the form of a real-time heart rate waveform to complement and reinforce the impact of the training. Usability testing (n=6) indicated that the overall response to the design and user experience of the system was perceived positively. All participants indicated that the system had a positive effect on stress management and that they would use it at home. Areas of improvement were identified, which focused primarily on the delivery of training and education on BNT through the app.

**Conclusions:**

The outcome of this project was a wearable sensor system to deliver BNT at home. The system has the potential to offer a complementary approach to blood pressure and stress management at home and reduce current accessibility barriers.

## Introduction

High blood pressure is one of the major global risks for mortality and the most important risk factor and leading cause of cardiovascular disease. It accounted for 7.5 million or 13% of all deaths globally in 2008 and affects approximately 30% of adults older than 25 years [ 1, 2]. High blood pressure also has an extremely high economic burden on health care systems, with an estimated cost of more than $370 trillion, representing 10% of the global health care expenditures [ 2]. Because of its impact, controlling high blood pressure has been stated as an international health priority [ 1].

The 2 primary approaches to managing blood pressure levels are lifestyle modifications (diet and exercise) and pharmacological treatments (antihypertensive drugs) [ 3]. However, additional interventions may be required when medication side effects are severe, lifestyle changes are not sufficient to lower or maintain blood pressure, or the patient prefers nonpharmacologic treatment [ 4]. Behavioral approaches offer an additional, complementary option for patients in these groups.

### Behavioral Neurocardiac Training

The Cardiac eHealth & Behavioural Cardiology Research Unit at Toronto General Hospital has developed an approach called “behavioral neurocardiac training” (BNT), which offers a complementary approach to managing stress and high blood pressure [ 5, 6]. BNT aims to improve the patient’s ability to recover from stressful stimuli and helps reduce unnecessary strain on their heart and blood vessels. Nolan et al [ 5, 6] demonstrated that BNT reduced daytime systolic blood pressure by 2.4±0.9 mm Hg ( *P*=.009) and 24-hour systolic blood pressure by 2.1±0.9 mm Hg ( *P*=.03). This reduction was confirmed to be independent of pharmacologic interventions. BNT was also found to reduce symptoms on the Perceived Stress Scale ( *P*=.001). BNT achieved these results by stimulating and exercising the autonomic reflexes, making them more efficient, and increasing total heart rate variability. As a result, vagal heart rate modulation was significantly increased, reducing the effect of stress and sympathetic activity on blood pressure [ 5, 6].

BNT is a multistep process that involves cognitive-behavioral therapy for stress management and the training of a paced breathing technique to regulate recovery from stress. Pacing breathing at 6-10 breathes per minute elicits respiratory sinus arrhythmia (RSA), where heart rate synchronizes with breathing and there is increased oscillations in heart rate. This increases total heart rate variability (HRV), exercising the autonomic nervous system’s reflexes and counteracting parasympathetic inhibition after stress [ 5, 6].

This breathing technique is reinforced using HRV biofeedback. Biofeedback is the process of gaining greater awareness of aspects of one’s physiology using monitoring equipment, with the aim of controlling it [ 7]. In BNT, the patient completes stress reactivity and recovery exercises that are complemented with real-time biofeedback. As they recover from stress using the paced breathing technique, monitors display a breathing pacer and HRV measures. These act as operant feedback to reinforce the patient’s progress toward a therapeutic goal [ 5, 6]. A clinician guides this process and helps the patient interpret the results.

### Limitations of Existing Solutions

BNT is currently limited to clinical settings because of two main difficulties. First, it requires a number of complex physiological monitoring devices, including a 12-lead ECG for data collection and a desktop computer running customized data visualization software. The monitoring devices are physically large and costly, presenting a significant barrier for widespread deployment of this technique. Second, the existing interface for displaying the biofeedback and breathing pacer was designed for use with clinician oversight. The clinician would guide the patient, direct their attention to the appropriate areas of the screen, and help interpret the meaning of the information being provided. Without this guidance, the user could find the interface to be technically complex to interpret independently. These issues create an accessibility barrier, preventing alternative delivery methods.

Existing commercial solutions that attempt to address these barriers include the emWave system (HeartMath Inc, Boulder Creek, CA, USA), which is a portable, consumer-focused system for biofeedback at home [ 8]. However, the emWave system measures heart rate using photoplethysmography, which is not as accurate as ECG, the standard reference signal for HRV. This is the reason why commercially available wrist-worn sensors such as the Fitbit Charge HR and Apple Watch cannot replace the ECG sensor, despite having a more user acceptable form. With photoplethysmography, R-peaks are not as evident, and sampling frequency is low, making it difficult to perform the precise HRV calculations required for BNT biofeedback. Another difficulty with the emWave system is that it focuses solely on paced breathing, whereas BNT is a multistep process that includes cognitive-behavioral therapy in addition to the paced breathing exercises. At this time, there are no commercial solutions that offer all the features of BNT for home use.

### Motivation and Objectives

Wearable wireless sensor systems represent a network of wearable computing devices that can be implanted or worn on the body to collect real-time physiological data [ 9, 10]. These sensor systems consist of sensor nodes to collect and transmit physiological information and an aggregator unit that acts as a central hub to fuse, visualize, and provide feedback on the data. Such systems offer an opportunity for the delivery of BNT outside of the clinic by providing patients with access to real-time data at home. This approach could possibly reduce accessibility barriers to care and may empower the patient by enabling self-care [ 11].

The intersection of behavioral approaches for blood pressure management and the technological domain of wearable wireless sensor systems has guided the development of this project. Our goal was to design, develop, and evaluate a wearable ECG sensor system for the delivery of BNT in a home setting. The system is intended for short-term use, whereby users take the system home and train on cognitive-behavioral therapy and paced breathing exercises through a series of sessions. Once trained, the goal is for users to be able to better manage their stress levels, without using the system.

This paper describes the design of *Beat*, a wearable sensor system that includes an ECG sensor and a mobile app, and discusses the results of the feasibility evaluation with focus on the usability of the system.

## Methods

This comprehensive project involved the design, development, and evaluation of the *Beat*sensor system. The system is intended for use in home settings, with adult patients with elevated blood pressure as the target audience. The development of the *Beat*was conducted in 3 major phases. Phase 1 and 2 included the development of the ECG sensor and BNT mobile app respectively. Phase 3 included the feasibility evaluation of the system.

### Phase 1: Electrocardiographic Sensor Requirements and Development Process

The *Beat*sensor was designed to capture and wirelessly transmit a user’s ECG data in real-time to the mobile phone. The primary design objectives were a small device footprint, less weight, and an exceptional user experience (usability). These are extremely important in making the sensor compact and unobtrusive. These attributes can also affect user experience and general comfort. Furthermore, a systematic literature review identified such features as the most important by users of wearable sensors. As per the user-centered approach, user preferences are a key influencer of whether the device gains acceptance among users and is actually used for its intended purpose [ 12, 13].

Technical objectives include good signal quality, long battery life, and user safety. Signal quality is an important requirement for accurate calculation and analysis of HRV. A long battery life allows for multiple uses without recharging, reducing disruptions to the user’s daily routine. Finally, as the device is patient worn and intended for home use, the safety of the user is of utmost importance.

The *Beat*sensor was developed following an iterative development process with several iterations of prototypes created and refined into the final design. Verification and validation testing were also conducted to ensure quality and that all user requirements were met. The development process was managed under the International Organization for Standardization (ISO) 13485 standard, which defines the quality management process for the design and manufacture of medical devices [ 14].

### Phase 2: Behavioral Neurocardiac Training Mobile App Requirements and Development Process

The BNT app was designed to guide the user through the BNT exercises and provide real-time HRV biofeedback. This required the app to communicate with the *Beat*sensor in real time and process data for HRV analysis. The key design objective was to have a patient-friendly interface for ease of use and user satisfaction. This is essential as the system is intended for use in home settings, without any clinical guidance. The interface must be able to guide the user and help them interpret what is happening. Furthermore, the app must be able to process ECG data and provide HRV biofeedback in real time, as required by BNT.

The BNT app was developed on the BlackBerry 10 mobile platform, as it provided Bluetooth Low Energy support and more control over connection parameters, compared to other mobile platforms. Development followed an iterative fashion using the Agile software methodology [ 15]. Verification and validation testing were also conducted, and the project was managed under the ISO 13485 standard.

### Phase 3: Feasibility Study

The purpose of the feasibility study was to evaluate usability, user interaction, and satisfaction with the sensor system to identify areas of improvement. These were accomplished through usability testing, observations, and qualitative questionnaires.

The feasibility study was conducted at the Cardiac eHealth and Behavioural Cardiology Research Unit at Toronto General Hospital in Toronto, Canada, in July and August, 2014. Participants were adults aged between 24-74 years and were recruited through flyers posted at Toronto General Hospital. Those who volunteered attended a single 1-hour session at the hospital, where they tested the system and provided their feedback. During the session, participants were asked to complete 4 main tasks, which included (1) usability testing of the wearable sensor system (users were asked to set up, connect, and wear the system); (2) completing 1 lesson of the BNT app; (3) completing a paper-based usability questionnaire (17 questions with 5-point Likert scale, developed by the study team to identify participant perceptions and overall satisfaction with the system); and (4) debriefing interview (conducted by a study team member).

There was a sample size of 6 participants based on the fact that the majority of usability issues can be detected with 5 participants [ 16]. This experiment was approved by the Institutional Research Ethics Board (Ref No: 13-6888-AE).

## Results

The results of this study are presented following the same 3 phases outlined in the methodology section.

### Phase 1: Beat Wearable Electrocardiographic Sensor

**Figure 1:**
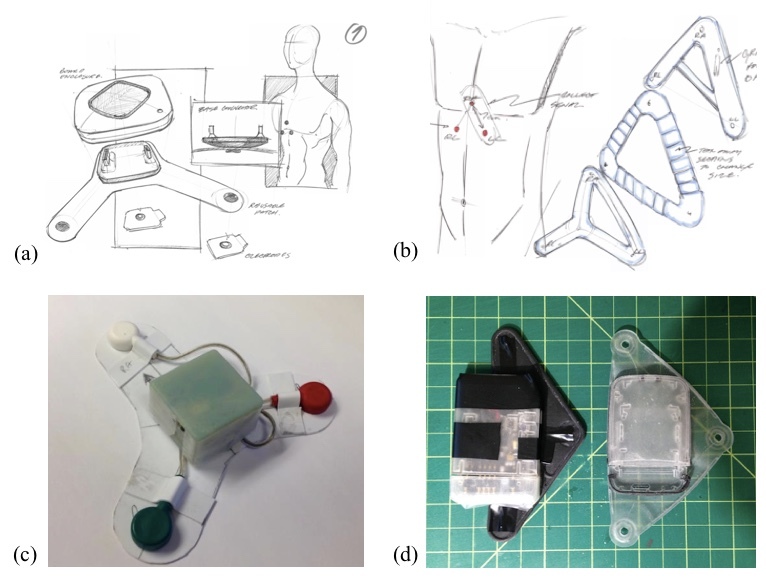
(a) Paper concept of modular design. (b) Paper concept of patch positioning. (c) Early functional prototype with vertical boards and flexible plastic. (d) Intermediate prototypes with horizontal boards and rubber patch.

The *Beat*sensor was developed as part of an iterative design process, as shown in Figure 1. Initial concepts were generated on paper ( Figure 1a,b), exploring different approaches for signal collection and body attachment. After several conceptual iterations, a patch design was chosen as it offers several advantages. First, the patch allows the electronics, signal collection, and body attachment components to be condensed into a small, compact package. This allows for a device with a small footprint and less weight that can be as unobtrusive as possible. Second, a patch was expected to be easy for users to wear, as a user only has to snap on electrodes and place the single unit on their chest, without having to place individual electrode locations. Finally, the patch allowed for the use of standard ECG electrodes, which provide a stable skin–electrode interface to improve signal quality and reduce noise.

An early functional prototype of the patch ( Figure 1c) was developed using vertically stacked circuit boards to minimize footprint and flexible plastic to orient electrodes. This prototype successfully demonstrated signal collection and transmission, but had a high device profile and did not flex enough to the contour of the body.

Further iterations of the sensor ( Figure 1d) addressed these issues and improved the overall aesthetic of the design. Horizontally placed boards were used to reduce the device profile with only a small increase in footprint, allowing the sensor to be unobtrusive and worn under clothing. The modular design addresses safety requirement by preventing users from ever being directly connected to line power, as the charging port is only exposed when the electronics module is disconnected from the patch.

**Figure 2:**
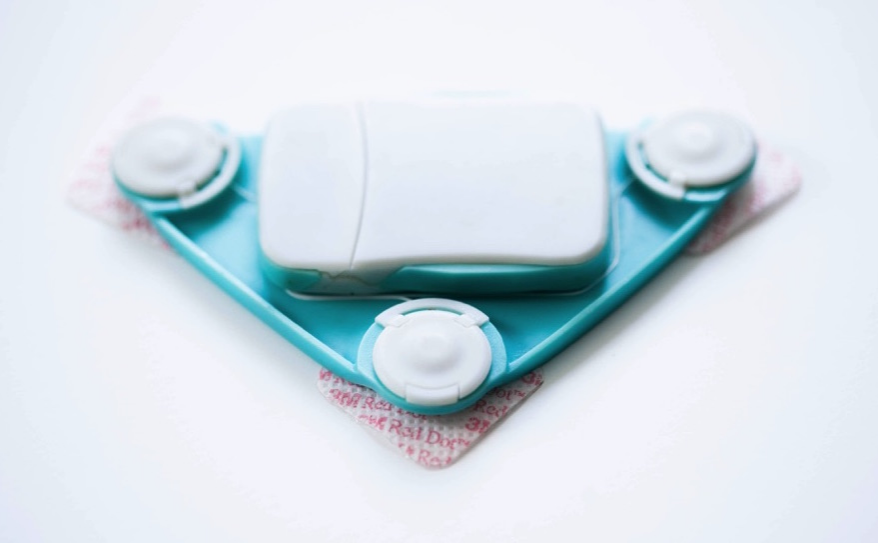
Final design of the Beat wearable ECG sensor with electrodes attached.

**Figure 3:**
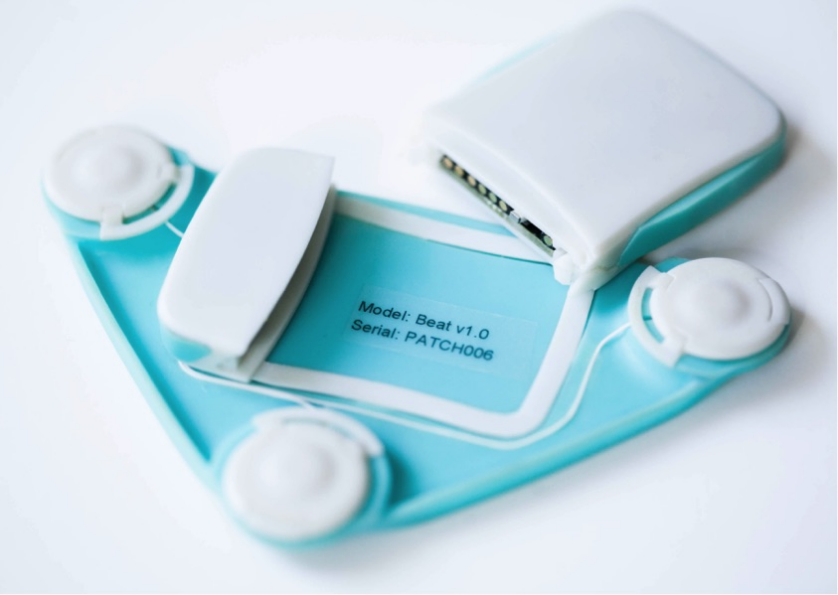
Individual components of the Beat wearable ECG sensor showing the electronics module and patch module.

The final design of the *Beat*sensor ( Figures 2and 3) is a modular patch with 3 components—electronics, patch, and electrodes. The electronics module houses and protects the sensor circuit and battery. Parts selection focused on components with low power consumption and small physical footprints. The final design uses an analog front end (ADS1293; Texas Instruments Inc, Dallas, TX, USA) to capture ECG at 260 samples per second, which meets the minimum requirements for accurate R-peak detection that is required HRV analysis [ 17]. A Bluetooth Low Energy system-on-chip radio (CC2541; Texas Instruments Inc, Dallas, TX, USA) is used to transmit the data to a mobile phone. Finally, an 110 mAh rechargeable lithium polymer-ion rechargeable battery supplies power. This battery provides a balance between physical size and power capacity and allows ECG streaming for 3.5 hours, exceeding the length of a BNT session (1 hour). As a real-time sensing and streaming device, the sensor does not store any data and has no memory.

The patch module is in the shape of a triangle that places the signal collection electrodes horizontally across the chest wall, and the right leg drive electrode is placed in between and below to reduce noise. The signal-collecting electrodes are separated by 10 cm in the final design to balance ECG signal quality and device size. The electronics module connects to the on-body patch through a clip mechanism and uses metal contacts for signal transfer. In this project, the sensor uses three Red Dot Repositionable electrodes (Product # 2660; 3M Company, Maplewood, MN, USA) to collect the signal and adhere the device to the user’s body. These electrodes are designed for easy application and have a large conductive area and strong adhesion [ 18].

The enclosure is tapered with a low profile to be less obtrusive to the user. The main enclosure is composed of acrylonitrile butadiene styrene plastic and the on-body patch is composed of flexible rubber silicone. Acrylonitrile butadiene styrene plastic offers excellent impact resistance and has less weight, whereas flexible silicone rubber is an inert material that is familiar to users and can be easily cleaned [ 19].

**Figure 4:**
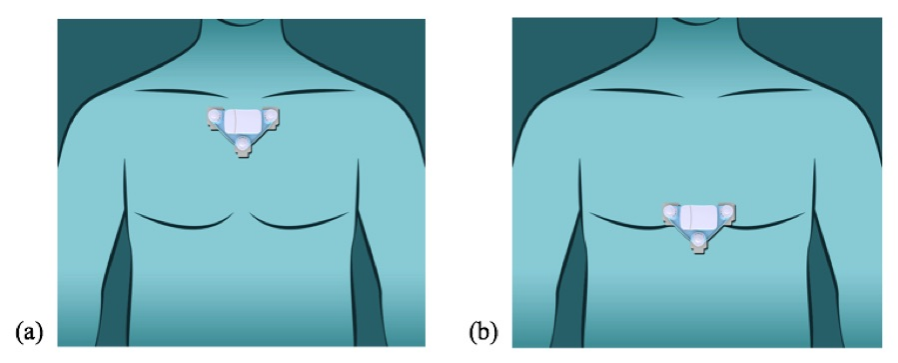
(a) Primary patch placement for lead I ECG. (b) Secondary patch placement.

The sensor is designed as a simple repositionable device that can be worn on multiple occasions and be easily adjusted for user preference or clothing. Figure 4describes the recommended patch placement areas on the body. These are based on the standard lead I ECG electrode placement and research on electrode placement for single-lead applications [ 20, 21].

As part of the ISO 14385 development process, the sensor passed through full verification and validation testing. This was to ensure all user and nonfunctional requirements were met and that the sensor collected ECG data accurately at the required sampling frequency. This included testing on a YellowJacket ECG simulator (Advantage Medical Cables Inc, Coral Springs, FL, USA) to verify that the ECG signal was collected accurately. The collected signal was analyzed on a computer and compared to the expected signal. Test execution was manual, with all test scenarios passing successfully.

### Phase 2: Beat Behavioral Neurocardiac Training App

The *Beat*BNT app serves to coach and guide the user through the BNT lessons, while providing real-time HRV biofeedback. The app presents BNT as a series of 5 lessons. Each lesson gradually introduces the users to BNT and paced breathing, goes through cognitive-behavioral therapy for stress management, and provides opportunities to practice the paced breathing technique and review their performance. As the app is intended for home use, it essentially takes the place of the clinician and guides the user through exercises using audio recordings, on-screen text, and interactive components.

**Figure 5:**
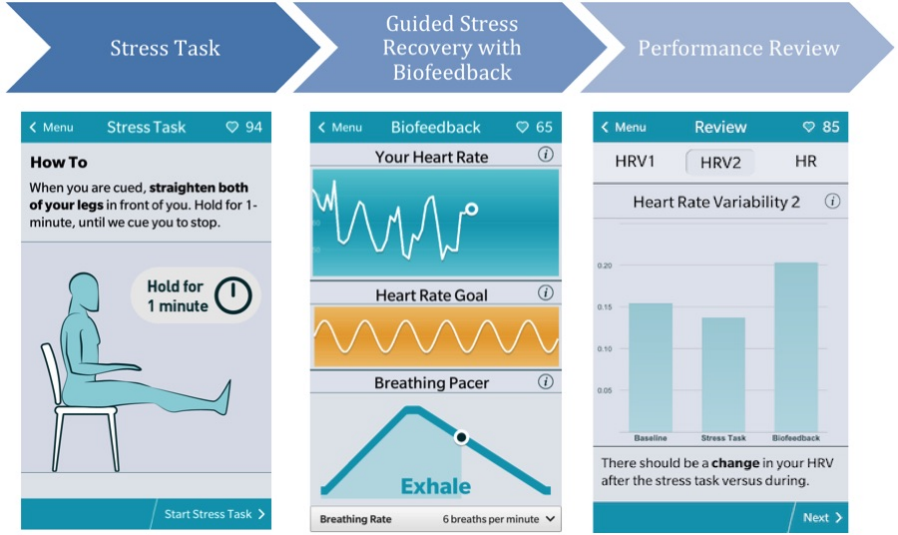
The user interface of the BNT application during each stage of the stress reactivity and recovery protocol.

The core exercise completed during each lesson is a stress reactivity and recovery protocol. It is used to reinforce the impact of the breathing technique and build the user’s skill in maintaining a slow, controlled breathing rate. There are 3 main components—stress task, guided stress recovery with HRV biofeedback, and performance review. Screenshots of the user interface for each of these components are displayed in Figure 5.

#### Stress Task

As part of the BNT exercises, users are exposed to mild-to-moderate stress tasks to engage physical or emotional arousal before they are guided in recovery using biofeedback. As with the existing in-clinic BNT protocol, the app uses a combination of physical and cognitive stress tasks that include seated leg raise, fist clench, serial addition, serial subtraction, and serial letters. Physical stress tasks are accompanied with real-time plots of HRV to highlight the impact of stress on heart activity. Cognitive stress tasks are adapted from the standardized cognitive tests and use a set of multiple-choice questions to test the user’s working memory, attention, math abilities, and pattern recognition to induce mild-to-moderate stress. For each stress task, the user is presented with instructions and is oriented to complete the task for 1 minute. With each lesson, the stress tasks increase with difficulty to counteract the training effect.

#### Guided Stress Recovery With Heart Rate Variability Biofeedback

Once the users have been exposed to stressful stimuli, they are guided in stress recovery using the biofeedback interface. This interface provides the users with real-time biofeedback while guiding them in maintaining a slow, controlled breathing rate. As the users pace their breathing rate, the interface attempts to demonstrate the action–reaction perspective, where the user performs the breathing exercises (action) to elicit increased HRV (reaction or target outcome).

The main form of biofeedback used in the app is heart rate, derived in real-time from ECG captured by the *Beat*sensor. The heart rate waveform acts as a form of operant feedback, providing users with a real-time view of their physiology and how it is changing during the breathing exercise. When the users pace their breathing correctly, they see a quasi-sinusoidal heart rate waveform owing to the effect of the RSA. The biofeedback interfaces displays both a 1-minute window of real-time heart rate and a goal waveform demonstrating the target pattern and asks users to visually compare them to see progress toward the goal. The interface also presents a breathing pacer that can be adjusted to a specific breathing rate. It provides visual cues to the user on when they need to inhale, exhale, and hold breath in order to match the set breathing rate. This helps guide the users in slowing their breathing rate and maintaining a steady rhythm. The pacer serves only a demonstrative purpose and does not display the user’s actual breathing rate.

#### Review

Once the users have completed stress recovery, the app displays review screens that present an overall view of their performance during the exercise. Performance is measured by heart activity in the form of heart rate and time domain HRV measures. The performance reviews compare the measures both within and between lessons to highlight the impact of the breathing technique over time. The within-lesson review attempts to highlight the effect of stress tasks on heart activity and the effect of paced breathing and biofeedback in recovery.

#### Software Algorithm Testing

Verification, integration, and validation testing were also conducted for the app. This was to ensure that the app communicated with the sensor, processed data accurately, and presented BNT to the user as intended. Tests were conducted manually, with all scenarios passing successfully. The QRS (heartbeat) detection algorithm was also rigorously evaluated against the MIT-BIH standard ECG database. It yielded a sensitivity of 99.11% and positive predictivity of 98.82%, which is comparable to most high-performing QRS detection algorithms. The HRV analysis algorithms were tested and found to be comparable with the Kubios HRV software (University of Eastern Finland, Kuopio, Finland), a validated tool for this type of analysis.

### Phase 3: Usability Study Results

A total of 6 participants completed the usability test with the *Beat*sensor system. There were 2 male and 4 female participants. The study had 1 participant in the 25-34 years age range, 2 in the 45-54 years range, 2 in the 55-64 years range, and 1 on the 65-74 years range. Participants’ mobile phone use varied from no use at all (2/6) to using a mobile phone more than once a week (3/6), with the majority (4/6) finding mobile phones easy to use.

Observations and feedback were analyzed to identify issues with the sensor and the application, which were then prioritized by frequency and severity. The usability questionnaire results were compiled to get an understanding of user experience and satisfaction.

#### Findings

The testing identified no major issues with the *Beat*sensor, only minor issues that can easily be mitigated with simple adjustments to the design or training. Some of the participants were unsure what orientation to wear the sensor and where to place it. Participants also found it difficult to reset or power cycle the sensor. Finally, participants experienced some discomfort removing the sensor due to the adhesive electrodes pulling on hair. The issues are summarized in Table 1.

The primary issue identified with the BNT app was an inadequate level of training and education on core concepts of BNT. As a result of poor organization and presentation of information in these sections, users demonstrated a lack of understanding of those concepts. This resulted in several participants performing the exercises but being unable to fully interpret the results. Thus, they could not benefit from operant feedback and were sometimes unsure if they met their goal. Another issue was information overload on certain pages. This was a result of the user being asked to pace their breathing, view the biofeedback, and follow audio instructions, all at the same time. The issues are summarized in Table 2.

**Table 1:** Major issues identified with the *Beat*sensor during the usability study.

Issue #	Participants	Task	Issue
S1	P1, P5, P6	Wearing Sensor	Better guidance and training is required on how and where to wear the sensor. Wearable systems such as *Beat*should ensure that users are well trained and guided through the set up process, as they are not used to the technology being used.
S2	P1, P4	Resetting Sensor	Lack of defined controls to turn the sensor on and off caused confusion when troubleshooting issues. Simplifying a system can have consequences on intuitiveness. Therefore, designers must either make the controls salient to the users or incorporate breadcrumbs on the design, guiding the users through the process.
S3	P1, P3	Removing Sensor	Adhesive electrodes cause discomfort to users on removal. Designers of future wearable devices should keep in mind the balance between function and user perception.

**Table 2:** Major issues identified with the BNT app during the usability study.

Issue #	Participants	Task	Issue
A1	P1, P2, P4	Education	Poor organization and presentation of information resulted in a lack of understanding of core concepts (HRV ^a^, RSA) required to interpret key sections of the app (Biofeedback, BNT, performance reviews).
A2	P1, P4, P5	Biofeedback	Biofeedback mechanism (heart rate and goal) was not always clear. Users were unsure of what to expect and whether they were completing the exercise correctly. Future interventions should consider a simpler and more intuitive interface that does not require users to comprehend complex medical terminology.
A3	P1, P4, P5	Review	Interpreting the performance review was difficult. It was not immediately obvious whether user results match the goal. Similarly, users should be presented with a simple display of success, providing cues on how to improve the next time.
A4	P4	Biofeedback	Biofeedback screen with audio creates information overload. User attention is divided between paced breathing, heart rate wave, and audio instructions on stress countering steps.

^a^BNT: behavioral neurocardiac training, HRV: heart rate variability, RSA: respiratory sinus arrhythmia.

#### User Experience and Satisfaction

Overall, users’ perceptions of the design and usability of the system were very positive. Participants found the sensor to be unobtrusive, reporting that it was compact and small and comfortable to wear and that it did not negatively affect day-to-day tasks. Participants also indicated that they would not hesitate to wear the sensor and that they did not experience any negative reactions while wearing it. However, a few participants indicated that the sensor might attract attention. Overall, the system was reported as easy to use, with high user satisfaction (4 of 6 of users were very satisfied).

In the postsession debrief interview conducted by the study coordinator, all participants stated that the system had a positive effect on their stress management, that they would use such a system at home, and that they would use this system if it were available to them. Participants noted that the sensor was easy to use, convenient, and comfortable to wear.

## Discussion

### Beat Wearable Electrocardiographic Sensor

The 3 key objectives that guided the design process for the sensor were small device footprint, less weight, and a patient-friendly interface. These were identified as the most important requirements and key influencers of device acceptance on systematic literature reviews [ 12, 13]. The results of this project indicate that the *Beat*sensor has met these objectives, which is reflected in the high user satisfaction.

All participants noted the sensor to be compact and small, and the majority of participants stated that wearing the sensor would not affect day-to-day tasks. This indicates that the sensor met its core objectives. However, it should be noted that half of the participants stated that the device attracted attention. This is possibly a result of the setup of the test and may not be representative of the design of the sensor. During the testing sessions, the sensor was kept visible to ensure it was functioning correctly. However, in the intended final use case, the participant would wear the device under their clothing and cover it up, thus making it less likely to attract attention.

The results of the study also indicated that the device was found to be well accepted with the limited sample. The majority of participants found the sensor to be comfortable and easy to use, with no negative reactions. Furthermore, the majority of participants stated that they would not hesitate to wear the device. This lack of hesitation and minimal burden on day-to-day tasks indicates that the sensor was found to be unobtrusive to users and that they would be interested in using the sensor in a home setting. This is important to gain user acceptance in use cases where long periods of monitoring are required.

These results indicate that the *Beat*sensor is a potentially viable candidate for remote patient-monitoring applications. It was comfortable and discreet, with participants having no issues wearing the sensor during the testing sessions. However, there are a few areas of improvement that should be addressed in future versions of the device.

The electrodes were found to cause discomfort when removed from skin with hair. Capacitive electrodes that collect a signal without direct skin contact or textile electrodes embedded in clothing offer some alternatives. However, these require some alternative form of adhesion to the skin to function. As this issue only affects a subset of users during sensor removal, the best possible course of action based on system characteristics that have driven the design would be to maintain the current design and focus on alternative placement options.

As currently designed, the sensor requires the application to provide guidance on how to orient the sensor on the body. Moving forward, the design of the sensor enclosure should provide users with some indication on how to place the sensor with the use of guiding labels, diagrams, and markers. This offers flexibility to users and caters to both beginner and expert users. Furthermore, it allows the sensor to be operated independently of the application, enabling other use cases.

The streaming nature of the device limited design complexity, but increased radio power consumption and limited the use of the sensor in the vicinity of a mobile phone. Future designs should incorporate on-board microcontrollers and flash memory to enable on-board processing of ECG, real-time alert notifications without a mobile phone, and storage of signals on board.

### Beat Behavioral Neurocardiac Training Application

The findings of the usability study demonstrate that the BNT application design was likely patient friendly and in alignment with our initial objectives. However, during testing, areas of improvement were identified, specifically around the presentation and organization of educational information and the biofeedback interface.

BNT requires the user to understand HRV and RSA in order to complete the exercises. This is important for the user to interpret the biofeedback and get the full benefit of the program. In usability testing, it was found that several participants had trouble understanding the meaning of HRV and RSA and had difficulty interpreting the information shown on the screen. Participants expressed that the education sections were slightly overwhelming because they presented a lot of information at once, without providing sufficient opportunities for review. Some participants also indicated that audio recordings were not conducive to covering the material and inquired about a text-only option. The inadequate level of training on HRV and RSA can be traced as the root cause of the issues whereby users had trouble interpreting the biofeedback and performance review screens.

To mitigate the issues with education and provide an improved user experience, the educational components need to be restructured and simplified to be more efficient in content delivery. Currently, the application delivers educational content with audio recordings and on-screen text, with each section generally 5 minutes long and covering multiple topics. To improve content delivery, the content can be broken into small, easily digestible modules that take less than a minute or two to complete. The module should provide a more interactive and graphical experience and reduce the reliance on audio recordings. Furthermore, the content needs to be reviewed to improve organization and language and clinically screened to ensure it is consistent with guidelines for delivering BNT in the clinic setting.

The biofeedback interface displays the user’s heart rate waveform to demonstrate the RSA pattern as a form of operant feedback. Users were expected to detect if their heart rate matched the provided goal heart rate waveform. Several participants faced issues interpreting the biofeedback screen and were unable to detect when they had achieved the goal. It may be useful to explore alternative display approaches such as configural displays to improve information saliency and promote the ability to see trends [ 22]. In configural displays, low-level data arrange in space to create higher order forms. This can potentially be applied to biofeedback interface design by mapping the main domain constraints (RSA, changes in HRV) to geometric forms. As the user completes the breathing exercises, the system states will change over time and emergent features may appear if the user is achieving the correct breathing goal.

### Limitations

The primary limitation of this study is that the user satisfaction questionnaires and interview responses could be influenced by response bias, specifically social desirability bias [ 23]. This is especially relevant as the interviewer was also involved in the development of the system and the effect of bias may inflate the positive responses to the system.

In addition, the evaluation was conducted in a controlled environment, which is not fully representative of the final intended user environment. Future testing should involve participants taking the system home, enabling the collection of user satisfaction data based on use in an actual home setting.

### Conclusions

In this project, the *Beat*wearable sensor system was developed for the delivery of BNT in home settings for stress and blood pressure management. A mobile app guides users through BNT exercises and provides real-time HRV biofeedback using a wearable ECG sensor. The system was evaluated in a usability study, in which the overall response to the design and usability of the system was very positive, with high user satisfaction. All participants indicated that the system had a positive impact on their stress management and that they would use it at home.

The findings of the study identified areas of improvement, specifically focusing on the presentation of information during the lessons. These findings can also be fed back into the in-clinic BNT protocol to improve user experience. New features for the ECG sensor were identified that would enable future applications in remote patient monitoring. Although further iterations and refinements are possible, this system is a step forward toward providing a complementary approach to blood pressure and stress management at home.

## Abbreviations

BNT: behavioral neurocardiac training
ECG: electrocardiography
HRV: heart rate variability
ISO: International Organization for Standardization
RSA: respiratory sinus arrhythmia
